# Addition of Vitamin A Intake Data during Compartmental Modeling of Retinol Kinetics in Theoretical Humans Leads to Accurate Prediction of Vitamin A Total Body Stores and Kinetic Parameters in Studies of Reasonable Duration

**DOI:** 10.1093/jn/nxz112

**Published:** 2019-06-12

**Authors:** Jennifer Lynn Ford, Joanne Balmer Green, Michael H Green

**Affiliations:** Department of Nutritional Sciences, College of Health and Human Development, The Pennsylvania State University, University Park, PA, USA

**Keywords:** dietary vitamin A intake, model-based compartmental analysis, theoretical humans, tracer kinetics, vitamin A assessment, vitamin A total body stores, WinSAAM

## Abstract

**Background:**

Mathematical modeling of theoretical data has been used to validate experimental protocols and methods in several fields.

**Objectives:**

We hypothesized that adding dietary vitamin A intake data as an input during compartmental modeling of retinol kinetics would lead to accurate prediction of vitamin A total body stores (TBS) at 2 specified study lengths and would reduce study duration required to accurately define the system.

**Methods:**

We generated reference values for state variables (including TBS and intake) and kinetic parameters for 12 theoretical individuals (4 each of children, younger adults, and older adults) based on modeling plasma retinol tracer data for 365 d. We compared TBS predictions using data to 28 d (children) or 56 d (adults) without and with intake included in the model to reference values for each subject. Then, by truncating data sets from 365 d, we determined the shortest study duration required to accurately define the system without and with inclusion of vitamin A intake.

**Results:**

Reference values for TBS ranged from 30 to 3023 µmol. Study durations of 28 and 56 d were sufficient to accurately predict TBS for 6 of the 12 subjects without intake; adding intake resulted in accurate predictions of TBS for all individuals. When intake was not included as a modeling input, durations of 35–310 d were required to define the system; inclusion of intake data substantially reduced the time required to 10–42 d.

**Conclusions:**

Inclusion of vitamin A intake as additional data input when modeling vitamin A kinetics allows investigators to accurately predict TBS and define the vitamin A system in studies of reasonable length (4 wk in children and 8 wk in adults). Because it is generally possible to obtain estimates/measures of intake, including such data increases confidence in model predictions while also making studies more feasible.

## Introduction

Since model-based compartmental analysis (1) was first applied to study vitamin A kinetics in rats (2), and then later in humans (3), investigators have recognized the importance of study duration in the accurate prediction of model parameters. For example, if vitamin A total body stores (TBS) are high, very long experiments (∼400 d) may be required (4). When a study is found to be too short for modeling to accurately define the true terminal slope of the tracer response curve, model-predicted vitamin A disposal rate (DR) will be overestimated, as will vitamin A intake, because modeling as typically applied to the vitamin A system assumes a steady state (i.e., functional input equals output). Importantly, when DR is overestimated, vitamin A stores will be underestimated (4), both at higher and at lower levels of vitamin A status.

In 2018, Gannon et al. (5) reported on the impact of experiment length on prediction of vitamin A stores and other parameters in 7 young women studied for 97–152 d. Based on truncating the data for each subject to various times, the authors showed that as study duration was increased, predictions of DR decreased and estimates of total traced mass (primarily vitamin A stores) increased. Also, by examining data for 1 additional subject studied for 6 y, they suggested that a duration of >200 d may be required to accurately estimate parameters of interest. Studies of this length may be difficult for reasons such as challenges with participant compliance, steady-state considerations, and limits of analytical detection. However, because compartmental analysis is increasingly recommended as a useful approach for estimating vitamin A status (6), it is important for researchers to have reliable guidance about study duration.

In related work, we recently showed (7) that by adding an estimate of vitamin A intake into the data input for modeling, we obtained physiologically reasonable predictions of intake and DR, and thus TBS, in older US and Chinese adults previously studied in 52-d kinetic experiments (8, 9). In contrast to values for DR calculated as in the original analysis (10) (12 µmol/d for 7 US subjects and 5.1 µmol/d for 6 Chinese subjects), which were reflected in unrealistic predictions of dietary vitamin A intake (16 and 7 µmol/d, respectively), the addition of an estimate of intake equivalent to the mean RDA for adults (2.8 µmol/d) during modeling resulted in DR predictions of 2.1 (US) and 2.2 µmol/d (Chinese) and 2.8 and 2.9 µmol/d, respectively, for intake. At the same time, estimates of TBS (2056 compared with 783 µmol for US subjects and 594 compared with 219 µmol for Chinese subjects) and “days of vitamin A stores” (981 compared with 64 d and 269 compared with 43 d, respectively) were higher using the new approach. These results led us to conclude that inclusion of vitamin A intake in the modeling data stream resulted in more reasonable estimates of vitamin A intake and DR, and thus more realistic predictions of TBS, when modeling results indicated that the original studies were of less-than-optimal duration. However, although the revised estimates were more physiologically reasonable, we could not evaluate the accuracy of the predictions because actual vitamin A stores had not been measured; the same limitation applies to the results of Gannon et al. (5) that were discussed previously.

Here, we asked whether inclusion of dietary vitamin A intake data as an additional input during compartmental modeling would improve the accuracy of model predictions of TBS and other state variables as well as retinol kinetic parameters and whether the additional data would impact the study duration required to obtain accurate predictions. To evaluate these hypotheses, we generated data for 12 theoretical human subjects with a range of values for age, state variables (including dietary vitamin A intake and TBS), and retinol kinetic parameters to use as reference values—an approach we have previously used to validate other methods in the vitamin A field (11–14). Our current results indicate that addition of vitamin A intake as a modeling input allows for accurate prediction of state variables, including TBS, and retinol kinetic parameters in studies of reasonable duration.

## Methods

### Theoretical subjects

Using methods similar to those described by Ford et al. (14), we postulated 12 theoretical subjects (**Supplemental Table 1**), 4 each of children, younger adults, and older adults. For each subject, we assigned physiologically reasonable values for relevant demographics and state variables (including vitamin A intake, plasma retinol pool size, and extravascular vitamin A) based on results adapted from previously published vitamin A studies in children (15–17), younger adults (5, 18–21), and older adults (7–9, 18, 22), as well as unpublished data (Supplemental Table 1). Then, parameters specified in the model described in the next section were assigned by adapting published values or choosing physiologically reasonable estimates.

### Compartmental model

**FIGURE 1:**
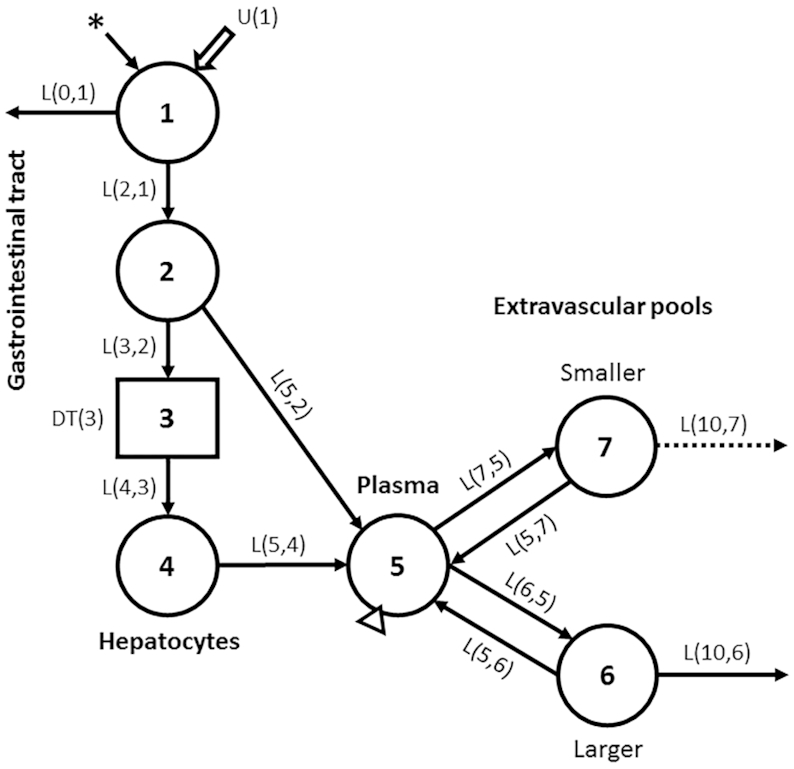
Compartmental model describing whole-body vitamin A metabolism in humans. Circles represent compartments, the rectangle is a delay element, and DT(3) is the delay time spent in component 3; interconnectivities between components (arrows) are fractional transfer coefficients [L(I, J)s, or the fraction of retinol in compartment J transferred to compartment I each day]. Compartment 1 is the site of introduction of ingested tracer (asterisk) and dietary vitamin A [U(1)]. Components 1–4 represent digestion, absorption, and chylomicron processing until uptake by hepatocytes (compartment 4), with subsequent secretion of retinol bound to retinol-binding protein into plasma compartment 5; compartment 5 is the site of sampling (triangle). Retinol in plasma can exchange with vitamin A in 2 extravascular pools (a larger compartment 6 and a smaller compartment 7); both are sites for irreversible loss from the system.

We articulated a 7-compartment model that describes whole-body vitamin A kinetics in humans (**Figure 1**). In this model, compartments 1–4 (including delay component 3) represent absorption and metabolism of dietary vitamin A until uptake by hepatocytes (compartment 4); compartment 1 is the site of input of both dietary and labeled vitamin A. From compartment 4, retinol bound to retinol-binding protein is secreted into plasma compartment 5; that retinol exchanges with vitamin A in 2 extravascular pools, a larger compartment 6 and a smaller compartment 7, which together represent extravascular vitamin A (TBS). Transfer between or out of compartments is indicated by fractional transfer coefficients [L(I, J)s, or the fractional transfer of retinol in compartment J to compartment I each day] and DT(3) represents the delay time in component 3. Initially, model simulations included loss from both compartment 6 and compartment 7, as shown in Figure 1 and as presumably occurs in vivo. However, because only plasma is typically sampled in human retinol kinetic studies (and this does not allow identification of the site or sites of loss), and because current results were only minimally affected if output was modeled from 1 compared with both extravascular compartments, we used a model with output only from compartment 6, as in a number of previous studies (3, 7, 17, 23).

### Generation of theoretical data for individuals

We populated the model shown in Figure 1 with the assigned kinetic parameters for each subject, including an absorption efficiency of 75%, the value used in several previous studies (10, 20). Then, following oral administration of labeled preformed vitamin A at time 0, plasma retinol tracer response data [fraction of dose (FD_p_)] were simulated from 3 h to 365 d; initial sampling times (3, 5, 8, and 12 h and 1, 2, 4, 7, 10, 14, and 21 d) were determined by geometric progression, with later points at 28, 35, 42, 49, 56, 70, 84, 114, 144, 200, 260, 310, and 365 d, totaling 24 sampling times. Tracer simulations were done using the Windows version of the Simulation, Analysis and Modeling software [WinSAAM version 3.3.0; http://winsaam.org; (24–26)]. Then, so that our theoretical data would be comparable to those collected in empirical studies, we added error with a normal distribution to the plasma data by using the RAND command in WinSAAM (27) and a fractional standard deviation (FSD) of 0.05. Model-generated FD_p_ data from 3 h to 365 d were plotted and fit to the model (Figure 1; but with loss from only compartment 6) for each individual. Then, weighted nonlinear regression analysis, using an FSD of 0.05 as a weighting factor, was performed to obtain model-predicted reference values for kinetic parameters for each subject. Finally, the assigned value for plasma retinol pool size [M(5); Figure 1, Supplemental Table 1] was fixed in the model for each subject and used in a steady-state solution in WinSAAM to calculate reference values for other compartment masses, including TBS, and transfer rates, including dietary vitamin A intake and DR.

Reference values for each subject were used as described in the following sections to evaluate our hypotheses that inclusion of vitamin A intake as a modeling input would *1*) lead to accurate prediction of TBS at 2 specified study lengths; and *2*) reduce study duration required to obtain accurate prediction of state variables, including TBS, and retinol kinetic parameters. Note that as in past uses of WinSAAM to model vitamin A kinetics, we assumed a steady state for our current analyses. This seems reasonable for well-nourished adults, even over 365 d, but it is less likely for young children who are growing and thus are presumably in positive vitamin A balance over that timeframe, assuming vitamin A intake is adequate. Although WinSAAM is able to accommodate growth and positive vitamin A balance, we assumed steady-state conditions for all of our subjects in order to keep this theoretical analysis as simple as possible.

### Impact of including dietary vitamin A intake as a modeling input on the accuracy of model-predicted TBS at 2 specific durations

To evaluate our first hypothesis, we selected a duration of 28 d for children because that is the time being used in ongoing field studies that are designed to assess vitamin A status in children in Bangladesh, Guatemala, Morocco, Nigeria, the Philippines, and Zambia; these experiments are being supported by the Bill & Melinda Gates Foundation and the International Atomic Energy Agency; a similar duration (35 d) was used in a previous vitamin A kinetic study done in this age group (17). For adults, the duration of 56 d is similar to the length of previous retinol kinetic studies that had been designed for analysis by compartmental modeling (8–10). For each subject, FD_p_ data to the appropriate time were plotted using WinSAAM and fit to the model (Figure 1; but with loss only from compartment 6) to obtain model predictions of TBS first without and then with vitamin A intake included in the modeling data stream. TBS results were compared to the reference value (discussed previously) for each subject; we specified that a value for TBS within 15% of the reference value would be considered accurate. Before testing, we fixed the values for the 2 model parameters that describe the initial absorptive phase [L(2, 1) and DT(3); Figure 1] at the reference values for each individual because these parameters are difficult to identify with precision and they have little influence on model predictions of postabsorptive kinetics and state variables; previous studies have taken the same approach (5, 7, 28). After fitting, iterations were performed using weighted nonlinear regression analysis, with an FSD of 0.05 as the weighting factor for plasma and, when applicable, for intake data, to estimate final values for the model parameters and their statistical uncertainty (25, 26). Then, we used the assigned value for plasma retinol pool size [M(5); Figure 1] for each subject in a steady-state solution in WinSAAM to estimate the compartment masses and transfer rates listed in the previous section.

### Determination of shortest study durations required to obtain accurate model predictions without and with dietary vitamin A intake included as a modeling input

To determine the shortest study length that provided accurate results without vitamin A intake added to the modeling data stream, we sequentially truncated each subject's data set from the full study length (365 d) and applied a similar analysis to that described in the previous section. Specifically, for each truncated data set, FD_p_ data were plotted compared with time and refit to the model (Figure 1; with loss only from compartment 6) using WinSAAM; for the adjustable model parameters, values were changed from the previous fit and iterations were performed. Model results were compared to reference values using the criterion specified previously for TBS as well as additional criteria (discussed later) that define the system. Here, with respect to modeling vitamin A, “to define the system” means that the structure and components of the proposed model accurately reflect the underlying physiology and kinetic processes that describe whole-body vitamin A metabolism (the system) as viewed from the plasma space. For this analysis, we specified that in addition to predicting TBS within 15% of the reference value, other state variables [e.g., dietary vitamin A intake, masses of vitamin A in compartments 6 and 7 (Figure 1), and DR] and retinol kinetic parameters would be considered accurate if within 25% of the reference values and the model's fractional transfer coefficients [L(I, J)s in Figure 1] should all have FSDs <0.5, a cutoff for parameter identifiability (25, p. 249). We specified less stringent criteria for defining the full vitamin A system than we did for only TBS because of the system's complexity (11 variables and parameters); in addition, we chose the more rigorous criterion for TBS because investigators are interested in quantifying stores as an index of vitamin A status. We also specified that values for L(10, 6), which is partially defined by the terminal slope of the plasma isotope response curve, must be a positive (nonzero) value because all theoretical subjects were designed to be consuming vitamin A and thus their data would never reach isotopic equilibrium when the terminal slope would indeed be zero. We added this latter criterion because if the data do not extend long enough to define the terminal slope (as might happen at shorter study durations), the slope of the curve might converge to zero. See **Supplemental WinSAAM Deck**, which shows the model code for 1 subject without and with vitamin A intake included as a modeling input.

Then, to test our hypothesis that inclusion of dietary vitamin A intake as an input in the modeling data stream would decrease the time required to accurately define the system, we added the reference vitamin A intake for each subject to the data set and repeated the process described in the preceding paragraph, starting with the study length identified for each subject without intake included in the model, until we found the shortest length required to accurately predict reference values.

To investigate how error in estimates of vitamin A intake might impact our results, we repeated the analysis described in the preceding paragraph at the shortest duration determined for each subject, using values for intake that were 50% and 150% of the reference value. Then, we compared resulting TBS predictions to values obtained when the reference intake was used.

### Data manipulations and statistics

Data were managed in Microsoft Excel. Figure 1 was created using Microsoft PowerPoint, and image quality was improved using Adobe Photoshop; all other figures were created using GraphPad Prism 7.0 for Windows. For evaluating whether the model accurately defined the system kinetics, parameter identifiability was evaluated based on the values for parameter FSDs calculated using WinSAAM (24).

## Results

### Reference values for theoretical subjects

Assigned values for subject characteristics as well as reference values for state variables and retinol kinetic parameters for each subject are presented in Supplemental Table 1. Vitamin A TBS (compartments 6 plus 7; Figure 1) ranged from 30 to 1104 µmol for the 4 theoretical children (aged 6 mo to 5 y), from 203 to 1038 µmol for the younger adults (aged 22–35 y), and from 449 to 3023 µmol for the older adults (aged 45–60 y). Days of vitamin A stores, calculated as TBS/DR, ranged from 77 to 716, 189 to 416, and 303 to 1254 for the 3 groups, respectively. Vitamin A intakes ranged from 0.53 to 4.4 µmol/d for the children, from 1.2 to 4.0 µmol/d for the younger adults, and from 1.7 to 3.6 µmol/d for the older adults (i.e., 152–1258, 343–1144, and 486–1030 µg retinol activity equivalents/d, respectively). These intakes compare with RDAs (micrograms of retinol activity equivalents per day) of 400 for infants aged 0–6 mo, 500 for children aged 7–12 mo, 300 for children aged 1–3 y, 400 for children aged 4–8 y, 900 for adult men >19 y, and 700 for adult women >19 y (29). Reference values for additional calculated kinetic parameters, including transit time, residence time, and recycling time and number (30), are presented in **Supplemental Table 2** for each subject, as is crossover time [i.e., the day on which retinol specific activity (fraction of dose per micromole) in stores became greater than that in plasma], an index of the time required for tracer in plasma to mix with that in stores.

### Accuracy of model-predicted TBS at 2 specific study durations

**FIGURE 2:**
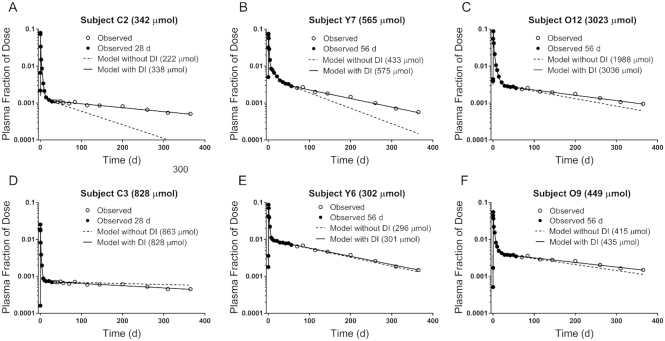
Theoretical observed plasma retinol tracer response data compared with time and model-calculated curves without and with the addition of dietary vitamin A intake as an input during compartmental modeling. Panels A and D show “observed” data for 2 theoretical children (C2 and C3, respectively; Supplemental Table 1) to 365 d as well as model simulations when data to 28 d were fit without and with intake included. Panels B and E show similar results for younger adults Y7 and Y6, respectively. Panels C and F show older adults O12 and O9, respectively, modeled to 56 d. Reference values for TBS are indicated, as are predictions without and with intake included. DI, dietary intake; TBS, total body stores.

We first evaluated the impact of adding dietary vitamin A intake as a modeling input on the accuracy of model predictions at the 2 specified study durations (28 d for children and 56 d for adults). **Figure 2** shows theoretical observed FD_p_ data to 365 d compared with model simulations for data fit to the 2 durations without and with intake included for 2 representative subjects from each age group; results for the other 6 subjects are presented in **Supplemental Figure 1**. For 3 of the subjects shown in Figure 2 (panels A–C), the terminal slope of the plasma tracer response curve was overestimated, as were model-predicted intake and DR, and TBS was underestimated when intake was not included. In contrast, when vitamin A intake was added to the modeling data stream, the model-calculated fits were visually similar to the curves obtained when the full data set (365 d) was modeled without intake (line not shown), and TBS was accurately predicted (within 15% of the reference value) for these subjects. For the other 3 subjects shown in Figure 2 (panels D–F), TBS was accurately predicted at the specified times even when dietary intake data were not included in the model. For all subjects (Figure 2, Supplemental Figure 1), TBS was underestimated for 6 of the 12 when dietary vitamin A intake was not included; when intake was added, TBS was accurately predicted at 28 d for all 4 of the children and at 56 d for the 8 adults (**Supplemental Figure 2**). In addition to TBS and model-predicted intake, only 2 retinol kinetic parameters [L(10, 6) and L(5, 6) in Figure 1] were noticeably impacted by the inclusion of diet in the model (Supplemental Figure 2). Overall, when vitamin A intake was included in the model, the specified durations (28 d for children and 56 d for adults) were long enough to provide both accurate predictions of TBS for all 12 subjects and model-calculated curves that agreed well with the fit to the full data set (365 d).

### Shortest study durations required for accurate model predictions without and with vitamin A intake included as a modeling input

**FIGURE 3:**
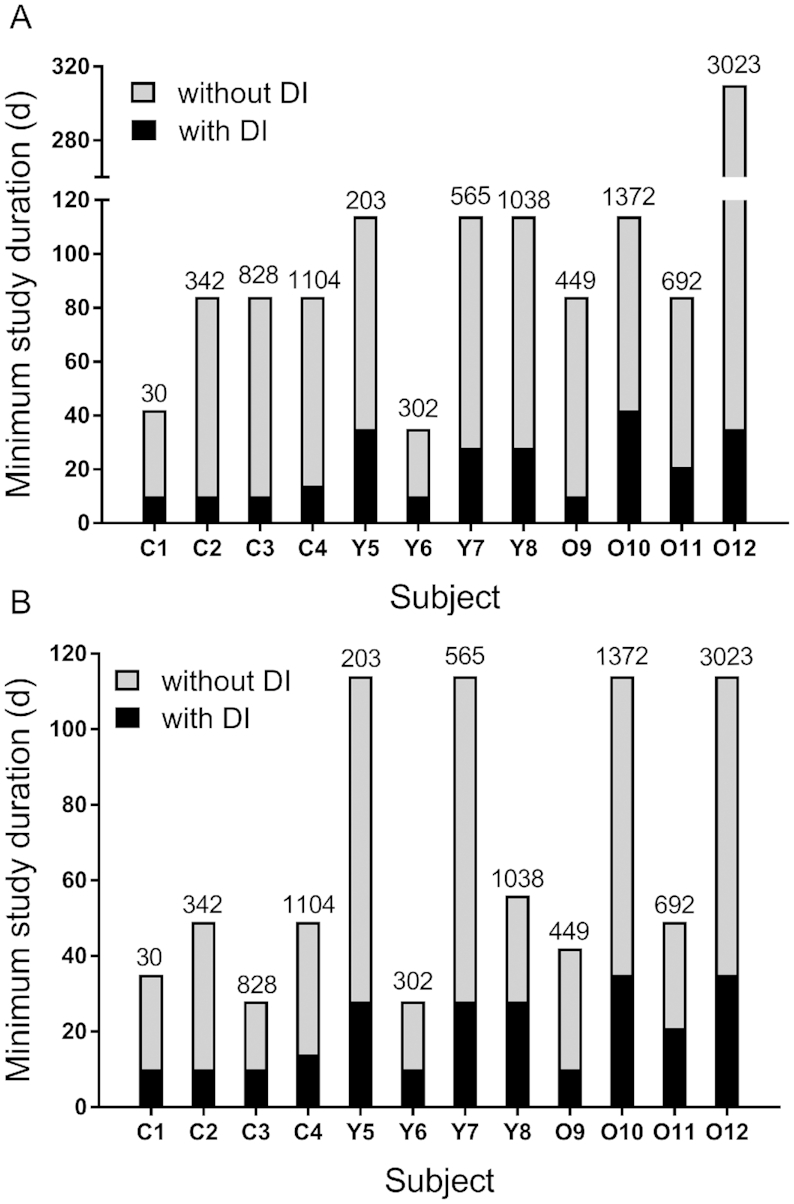
Shortest study length required for accurate prediction of state variables, including TBS, and retinol kinetic parameters (A) or only TBS (B) without or with dietary vitamin A intake included as an input during compartmental modeling. Reference values for TBS (in micromoles) are shown above the bars for each subject. Among the 12 theoretical human subjects, C1–C4 are children, Y5–Y8 are younger adults, and O9–O12 are older adults; see Supplemental Table 1 for more details about each subject. DI, dietary intake; TBS, total body stores.

Although the results described in the preceding section demonstrate that the specified times (28 and 56 d) provided accurate predictions of TBS when vitamin A intake was added to the model, it is possible that inclusion of diet would accurately define the system at even shorter study durations. Shown in **Figure 3** are results obtained by truncating the data sets to sequentially shorter lengths using the sampling times established for this work. When intake was not included as a modeling input (Figure 3A), required duration varied among individuals; the time required for accurate prediction of state variables, including TBS, and retinol kinetic parameters ranged from 35 to 310 d. In most cases, the time required for children was shorter (42–84 d) than that for adults (35–310 d). For the majority of subjects (9 of 12), the study duration required for accurate predictions by the model when dietary vitamin A intake was not included as a modeling input was either 84 or 114 d; TBS for these subjects ranged from 203 to 1372 µmol (Supplemental Table 1), and the crossover time (Supplemental Table 2) ranged from 11 to 20 d. Among the remaining 3 subjects, the 2 for whom required duration was shorter than 84 d (42 and 35 d) had TBS values of 30 and 302 µmol, respectively, and short crossover times (12 and 10 d, respectively); the subject who required the longest duration (310 d) had the highest TBS (3023 µmol) and highest value for crossover time (61 d).

When reference data for dietary vitamin A intake were added to the modeling data stream, the time required to obtain accurate predictions was reduced to 10–42 d (Figure 3A); this reflects a decrease ranging from 63% to 89%. In fact, state variables and kinetic parameters were accurately predicted for 50% of the subjects by 14 d, for 75% by 28 d, and for 100% by 42 d. With intake included in the model, the study length required to accurately define the system in the 4 children was only 10 or 14 d; accurate prediction for the adults required durations ranging from 10 to 35 d (younger adults) and from 10 to 42 d (older adults). It is also important to note that although data for our theoretical subjects were generated assuming a steady state even though growing young children are likely to be in positive vitamin A balance if intake is adequate, the study duration required for these children was short (10–14 d), and presumably any influence of positive vitamin A balance on model predictions during this time would be small.

**FIGURE 4:**
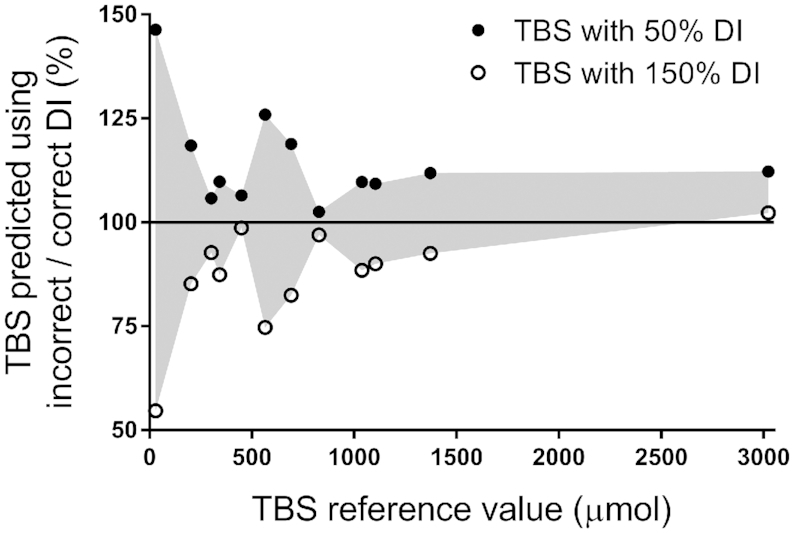
Impact of using values above or below the reference intake on model-predicted TBS for 12 theoretical human subjects. Shown are ratios of TBS predicted when dietary vitamin A intake was set at 50% or 150% of the reference value (“incorrect”) to TBS predicted using the (“correct”) reference value compared with each subject's reference value for TBS. The solid line indicates a ratio of 1. See also Supplemental Table 3. DI, dietary intake; TBS, total body stores.

Based on the shortest study duration required for accurate predictions in each subject (Figure 3A), we tested the impact of including values for vitamin A intake during modeling that were 50% or 150% of each individual's reference value. As illustrated in **Figure 4**, results indicated that TBS predictions for the majority of subjects (11 of 12) using the different values for intake were within 26% of the value predicted using the reference intake. For the other subject [the theoretical child with the lowest TBS (30 µmol)], predictions were within 46%, but this equates to an absolute difference in TBS of only 12 µmol. The predicted values for TBS when intake was varied, as well as the percentage change compared with the reference value for each subject, are presented in **Supplemental Table 3**.

Because most investigators are more interested in obtaining accurate estimates of TBS compared with the other variables and parameters evaluated here, we also determined required study durations without and with dietary intake included in the model when the only criterion was to predict TBS within 15% of the reference value. As illustrated in Figure 3B, in most cases (9 of the 12 subjects), without intake included in the model, the duration required to accurately predict TBS was shorter than that for predicting state variables plus kinetic parameters (Figure 3A). In fact, TBS was accurately predicted for all subjects in 114 d. When dietary intake was included, accurate predictions of TBS were obtained at the same study length as that needed to define the entire system to within our specified criteria for 10 of the subjects; for the other 2, the duration was 1 wk less.

## Discussion

The results of this study demonstrate that when vitamin A intake is included as a modeling input, study durations similar to those previously used for vitamin A kinetic studies (28 d for children and 56 d for adults) are long enough to accurately predict TBS and define the vitamin A system. In addition to improving the accuracy of model predictions, inclusion of vitamin A intake data during compartmental modeling can also dramatically shorten the study duration required (Figure 3). By using modeling to generate theoretical data, and then adding error to the data so data would be comparable to those collected in retinol kinetic studies in humans and calculating reference values for the parameters of interest, we established a “gold standard” for evaluating accuracy of predictions and were thus able to test our hypotheses. Theoretical data are used to test methods and evaluate protocols in pharmacokinetics (31), and we have previously used this approach to test other methods in the vitamin A field (11–14).

Although the idea of including steady-state information, such as vitamin A intake, as weighted data during compartmental analysis has been mentioned previously (25, p. 230), to our knowledge, this approach is not yet commonly used. In the case of vitamin A, the first application of this approach was in our work with Lopez-Teros et al. in 2017 (17), in which we added estimates of vitamin A intake obtained from assessment measurements made in Mexican children to constrain the terminal slope of the isotope response curve when modeling iterations converged the slope to zero and resulted in an underestimation of reported intake and the illusion of isotopic equilibrium. Our current results suggest that TBS reported for that group (823 µmol) in Lopez-Teros et al. (17) is likely accurate. More recently, as discussed previously, we applied the approach of including intake in the model to a different problem (7). Specifically, we realized in retrospect that originally published (10) model predictions for vitamin A intake and DR in older adults were unrealistically high (and thus model-predicted TBS was lower than if the predictions for those parameters had been more physiologically reasonable), presumably because the study length (52 d) was not long enough to fully define the terminal slope of the plasma isotope response curve for all subjects. By including estimates of vitamin A intake in a reanalysis of those data (7), we obtained more reasonable estimates of intake and DR as well as higher estimates of vitamin A TBS. As was true in the case of the work of Gannon et al. (5) described previously, the accuracy of the new estimates could not be evaluated. Our current work demonstrates the validity of the approach, which can now be applied retrospectively by others, as we did (7, 21).

Importantly, our finding that when the value used for vitamin A intake was reduced to 50% or increased to 150% of the reference value, estimates of TBS were within 26% for 11 of the 12 subjects (Figure 4) indicates that a good estimate of vitamin A intake, as we used elsewhere (7), should be adequate in most cases. In addition, it is worth mentioning that the use of dietary intake data as described here should be generalizable when modeling is applied to study the kinetic behavior of other nutrients.

In addition to the length of kinetic studies, another advantage of including an estimate of dietary vitamin A intake during modeling is that the number of blood samples collected from each subject can be reduced without compromising confidence in model predictions. For the theoretical subjects and sampling protocol used in our current work, 13–23 samples were needed to ensure accurate prediction of state variables (including TBS) and kinetic parameters when intake was not included. In contrast, when intake was added, 9–13 samples were enough, reducing the number of blood samples required by 4–11. Importantly, the capacity to reduce both study duration and sample burden on each subject has the potential to effectively lower cost, improve compliance, increase detection sensitivity, and lessen the probability that the steady state will be disrupted (e.g., by development of an infection).

In several previously published models for whole-body vitamin A metabolism that included 2 extravascular compartments (3, 17, 23), loss was modeled from the larger compartment (compartment 6; Figure 1), whereas in another study (5), it was modeled from the smaller extravascular pool (compartment 7). Presumably, loss occurs from many extravascular tissues in vivo, but without excretory data, one cannot identify the site(s) of loss and therefore it is lumped into a single output from 1 compartment during modeling. We found that results obtained when data to 365 d were fit to the model with loss from both extravascular compartments were similar to those determined using a model with loss only from compartment 6 (specifically, TBS predictions were within 5%), and thus we conclude that lumping of output for this system has little influence on model predictions.

Because compartmental analysis is increasingly used in conjunction with retinol isotope dilution (RID) equations to estimate TBS in individuals (14, 17, 32), we used our current data to gain insight into the impact of including vitamin A intake as an input during modeling on model-predicted RID coefficients. As discussed elsewhere (33, 34), coefficients are required as “correction factors” in RID equations in order to account for absorption and retention of the tracer dose (*Fa*) and isotope mixing with endogenous vitamin A pools (*S*). In the past, values used for the coefficients were those specified in an early application of the method (33), but they can also be obtained by using compartmental analysis (17, 21, 32, 34). For example, if a “super-person” approach (14) is used to develop a population model for a specific group using geometric mean FD_p_ data, then a population value for *FaS* at a specific time can be used to estimate TBS for individuals in that group. Here, to investigate the impact of including dietary vitamin A data on estimates of the composite RID coefficient *FaS*, we modeled the geometric mean FD_p_ data to 28 d for the group of theoretical children and to 56 d for the adults, without and with dietary intake included as a modeling input. Then, we determined *FaS* at times previously applied or suggested in RID studies [4 d (32), 7 d (14), and 14 d (35)] and compared those values to the reference values for *FaS* based on modeling the data sets to 365 d. When intake was not included, we found that geometric mean data were not sufficient to predict *FaS* within 15% of the reference value for any of the groups at any of the times. In contrast, when dietary intake data were added, *FaS* was accurately predicted for all 3 groups at all 3 times; see **Supplemental Table 4**, which also includes values for *FaS* based on modeling data for individual subjects. In addition, inclusion of dietary vitamin A intake during modeling resulted in a dramatic improvement in parameter identifiability (data not shown), especially for the parameter partially defined by the terminal slope [L(10, 6); Figure 1], which is critical for accurate prediction of TBS. Specifically, the coefficient of variation (FSD) for this parameter decreased by a mean of 81% across the 3 groups; the same trend was seen when the analysis was performed on individual subjects. These observations indicate that inclusion of intake data during modeling both increases confidence in the accuracy and reproducibility of model predictions, including TBS, and improves the accuracy of predictions of the composite RID coefficient *FaS*.

In conclusion, our results using theoretical subjects show that if vitamin A intake data are included as an input during compartmental analysis of retinol kinetic data, studies of relatively short duration are sufficient to accurately estimate TBS as well as other state variables and retinol kinetic parameters. Specifically, our current work indicates that a duration of 28 d would be a safe choice for vitamin A kinetic studies in children and that 56 d should be adequate for adults if intake data are included. These findings have important implications for future applications of compartmental analysis as a method for assessing vitamin A status and determining system kinetics because shorter studies with fewer plasma samples incur a lower cost and reduced subject burden and they are more feasible in children.

## Supplementary Material

nxz112_Supplemental_File

## Notes

This work was supported by the Bill & Melinda Gates Foundation (Project OPP1115464) and the College of Health and Human Development, The Pennsylvania State University.

Author disclosures: JLF, JBG, and MHG, no conflicts of interest.

Supplemental Tables 1–4, Supplemental WinSAAM Deck and Supplemental Figures 1 and 2 are available from the “Supplementary data” link in the online posting of the article and from the same link in the online table of contents at https://academic.oup.com/jn/.

Abbreviations used: DR, disposal rate; DT(I), delay time; FD_p_, fraction of administered dose in plasma; FSD, fractional standard deviation; RID, retinol isotope dilution; TBS, total body stores; WinSAAM, Windows version of the Simulation, Analysis and Modeling software.
