# Supplementary material for: Addition of Vitamin A Intake Data during Compartmental Modeling of Retinol Kinetics in Theoretical Humans Leads to Accurate Prediction of Vitamin A Total Body Stores and Kinetic Parameters in Studies of Reasonable Duration
Source: J Nutr. 2019 Jun 12;149(11):2065–72. doi: 10.1093/jn/nxz112 (PMC6825818; doi:10.1093/jn/nxz112)
Supplement: nxz112_Supplemental_File [file nxz112_supplemental_file.pdf]

Supplemental Table 1 Assigned values for demographics plus reference values for state variables and retinol kinetic parameters for 12 theoretical subjects[1]

Group	Children				Younger adults				Older adults			
Subject ID	C1	C2	C3	C4	Y5	Y6	Y7	Y8	O9	O10	O11	O12
Demographics												
Age, y	0.5	1	3	5	30	24	22	35	50	45	55	60
BW, kg	8	11	13	20	50	65	60	80	45	75	70	68
$[ROH]_p$, µmol/L	0.8	1.2	1.3	1.1	1.25	1.5	1.4	1.8	1.35	1.65	1.2	1.75
State variables												
U(1), µmol/d	0.5284	1.098	1.541	4.391	1.221	2.080	3.994	3.324	1.661	3.644	3.040	3.214
M(5), µmol	0.3200	0.6600	0.8450	1.100	2.175	4.240	3.650	5.570	2.640	5.380	3.650	5.180
M(6), µmol	27.85	333.8	800.9	1052	138.4	278.0	484.8	940.7	407.3	1151	664.4	2768
M(7), µmol	2.534	7.918	27.52	52.12	64.43	24.33	80.18	97.72	41.67	221.6	27.18	255.2
TBS, µmol	30.38	341.7	828.4	1104	202.9	302.3	565.0	1038	449.0	1372	691.6	3023
DR, µmol/d	0.3964	0.8236	1.156	3.294	0.9158	1.561	2.996	2.494	1.246	2.734	2.281	2.411
Days of stores	76.65	414.94	716.4	335.2	221.5	193.7	188.6	416.4	360.3	502.0	303.2	1254
Kinetic parameters												
L(2,1), d^{-1}	28.34	43.58	37.18	39.03	28.30	33.16	60.52	50.94	46.2	49.55	28.38	62.93
L(0,1), d^{-1}	9.438	14.5	12.38	13.00	9.422	11.04	20.15	16.96	15.39	16.50	9.450	20.96
L(5,2), d^{-1}	0.2934	0.4764	0.2133	0.4049	0.2885	0.4172	0.8233	0.5393	0.5007	0.5528	0.2812	0.6766
DT(3), d	0.2559	0.1862	0.1469	0.2008	0.2255	0.2436	0.1397	0.1743	0.2343	0.1813	0.1939	0.2176
L(5,4), d^{-1}	2.156	2.663	3.239	1.439	1.687	2.008	0.9169	1.099	1.964	0.6821	1.241	0.6081
MST_{RBP}, h	18.96	14.58	12.22	22.73	21.33	19.24	30.32	26.96	18.88	40.51	25.69	45.45
L(6,5), d^{-1}	2.666	6.900	30.03	22.53	2.059	4.087	2.838	3.508	4.766	5.632	2.763	5.587
L(5,6), d^{-1}	0.01640	0.01117	0.03024	0.02042	0.02573	0.05673	0.01519	0.01812	0.02783	0.02395	0.01174	0.009585
L(7,5), d^{-1}	12.56	10.1	39.85	21.72	9.243	9.037	3.812	3.490	15.47	5.529	3.061	4.937
L(5,7), d^{-1}	1.586	0.8420	1.223	0.4583	0.3120	1.574	0.1735	0.1989	0.9800	0.1342	0.4111	0.1002
L(10,6), d^{-1}	0.01423	0.002467	0.001444	0.003131	0.006615	0.005615	0.006180	0.002651	0.003060	0.002376	0.003433	0.0008711

[1]Values for the demographics age, body weight (BW), and plasma retinol concentration {$[ROH_p]$} were assigned based on information in the literature; see specifics for each group below. State variables include dietary vitamin A intake [U(1); Figure 1], compartment masses [M(I)], total body stores [TBS = M(6) + M(7)], disposal rate [DR = L(10,6) × M(6)], and days of vitamin A stores calculated as TBS / DR. Plasma retinol pool size [M(5)] was calculated using the assigned values for $[ROH]_p$ and BW as $[ROH]_p$ (µmol/L) × estimated plasma volume (L), where plasma volume was estimated as BW (kg) × 0.05 L/kg for the 4 children (Supplemental Reference 1) and BW (kg) × 0.045 L/kg for the 8 adults (Supplemental Reference 2). Kinetic parameters are fractional transfer coefficients [L(I,J)s] or fraction of retinol in compartment J transferred to compartment I each day; the delay time in component 3 [DT(3)]; and mean sojourn time to retinol-binding protein (MST_{RBP}), or the mean of the distribution of times for a retinol molecule entering compartment 1 to arrive in plasma compartment 5 bound to retinol-binding protein, calculated as $[L(2,1)^{-1} + L(3,2)^{-1} + DT(3) + L(5,4)^{-1}] \times 24$. For compartmental analysis, L(3,2) = L(2,1); DN(3) = 8 where DN is delay number; L(4,3) = 1; and L(5,2) was generated to be ~1% of L(2,1) (28). The model is shown in Figure 1.

Following are notes on information used to generate demographics, state variables, and kinetic parameters for the 3 groups of theoretical subjects:

For the 4 children: C1: low vitamin A intake with low/marginal vitamin A status adapted from (15,16,Supplemental Reference 3); C2: adequate intake (29) and adequate status using information from (16); C3: composite based on a group of Mexican children with adequate vitamin A intake and adequate/high vitamin A status (17); C4: high intake [above the upper limit for this age (29)] with high vitamin A status using results adapted from a group of Filipino children (Marjorie J. Haskell and Reina Engle-Stone, University of California, Davis; personal communication) as well as Mexican children of preschool-age (Veronica Lopez-Teros, Universidad de Sonora, Mexico; personal communication); retinol kinetics for these groups were analyzed using compartmental analysis (Jennifer L. Ford and Michael H. Green, The Pennsylvania State University; unpublished results).

For the 4 younger adults: Y5: low vitamin A intake that maintained low/marginal status adapted based on US and Bangladeshi adults studied in (18,19); Y6: composite based on a group of previously-studied European young adults with moderate intake and adequate status (Supplemental Reference 4,20) for which kinetics were adapted from (21); Y7: composite based on a group of US young adult women with adequate vitamin A status and higher-than-average intake (5); S8: based on a US adult woman studied for 6 y (5) for whom tracer data [Figure 5A in (5)] were extracted and compartmental analysis was applied (Jennifer L. Ford, The Pennsylvania State University; unpublished results).

For the 4 older adults: O9: moderate vitamin A intake and adequate status based on Bangladeshi adults studied in (22); O10: average intake and adequate status adapted from one US adult male subject (18) whose kinetic data were analyzed using compartmental analysis (Michael H. Green and Jennifer L. Ford, The Pennsylvania State University; unpublished results); O11 and O12: composites based on older Chinese adults with adequate vitamin A intakes and adequate vitamin A status (O11) and older US adults with high vitamin A status (O12) originally studied in (8,9) and later analyzed in (7) using compartmental modeling.

Supplemental Table 2 Reference values for additional kinetic parameters calculated for 12 theoretical subjects[1]

Group	Children				Younger adults				Older adults			
Subject ID	C1	C2	C3	C4	Y5	Y6	Y7	Y8	O9	O10	O11	O12
Parameter												
$\bar{t}(5)$, d	0.06568	0.05882	0.01431	0.02260	0.08848	0.07620	0.1504	0.1429	0.04942	0.08960	0.1717	0.09502
$\bar{t}(6)$, d	32.64	73.30	31.56	42.45	30.91	16.04	46.80	48.14	32.37	37.98	65.88	95.63
$\bar{t}(7)$, d	0.6307	1.188	0.8174	2.182	3.205	0.6351	5.762	5.028	1.020	7.448	2.432	9.980
$\bar{T}(5,5)$, d	0.8073	0.8014	0.7308	0.3339	2.375	2.717	1.218	2.234	2.118	1.968	1.600	2.148
$\bar{T}(6,5)$, d	70.26	405.3	692.6	319.4	151.2	178.1	161.8	377.2	326.8	421.0	291.3	1148
$\bar{T}(7,5)$, d	6.394	9.614	23.80	15.82	70.36	15.59	26.76	39.19	33.44	81.05	11.91	105.9
$\bar{T}$(SYS), d	77.46	415.7	717.1	335.5	223.9	196.4	189.8	418.6	362.4	504.0	304.8	1256
$\nu(5)$	11.29	12.62	50.06	13.78	25.84	34.65	7.102	14.63	41.86	20.96	8.319	21.61
$\bar{t}\bar{t}(5)$, d	6.789	32.87	14.31	24.34	8.572	5.589	26.55	28.46	8.606	23.94	36.44	58.02
CO, d	12	14	11	17	34	10	27	29	19	35	20	61

[1]Shown are reference values for kinetic parameters calculated using results from the model (Figure 1) fit to the full dataset (365 d) without inclusion of dietary vitamin A intake for theoretical children, younger adults, and older adults (n=4/group; see Supplemental Table 1). Calculated parameters include: transit times [$\bar{t}$(J), or the mean of the distribution of times a retinol molecule spends in compartment J during a single transit, calculated as 1/∑L(I,J)s exiting compartment J] for retinol in plasma [$\bar{t}(5)$] and in extravascular compartments 6 [$\bar{t}(6)$] and 7 [$\bar{t}(7)$]; mean residence times [$\bar{T}$(I,J), or the mean of the distribution of times the tracer spends in compartment I after entering the system via compartment J, where J is compartment 5 for tracer that was absorbed] for retinol in plasma [$\bar{T}(5,5)$], the extravascular compartments 6 and 7 [$\bar{T}(6,5)$ and $\bar{T}(7,5)$, respectively], and the system [$\bar{T}$(SYS), equal to $\bar{T}(5,5)$ + $\bar{T}(6,5)$ + $\bar{T}(7,5)$]; recycling number for plasma {$\nu(5)$, or the mean number of times a molecule of retinol recycles to plasma before being irreversibly lost, calculated as [$\bar{T}(5,5)$ / $\bar{t}(5)$] – 1}; recycling time to plasma {$\bar{t}\bar{t}(5)$, calculated as [$\bar{T}(6,5)$ + $\bar{T}(7,5)$] / $\nu(5)$}; and crossover (CO; day on which retinol specific activity in stores becomes greater than that in plasma, where specific activity = fraction of dose in plasma or stores / mass of vitamin A in plasma or stores, respectively). For more information on calculation of parameters, see (29).

Supplemental Table 3 Vitamin A TBS predicted using different values for dietary vitamin A intake at the shortest study duration determined for theoretical subjects[1]

Subject	Duration (d)	TBS (µmol) using reference value DI	TBS (µmol) using 50% DI (% Δ)	TBS (µmol) using 150% DI (% Δ)
C1	10	27	39 (44)	15 (–44)
C2	10	308	338 (9.7)	270 (–12)
C3	10	856	878 (2.6)	830 (–3.0)
C4	14	980	1071 (9.3)	882 (–10)
Y5	35	197	233 (18)	168 (–15)
Y6	10	306	324 (5.9)	284 (–7.2)
Y7	28	558	703 (26)	417 (–25)
Y8	28	1069	1173 (9.7)	946 (–12)
O9	10	411	438 (6.6)	405 (–1.4)
O10	42	1437	1607 (12)	1330 (–7.4)
O11	21	616	733 (19)	508 (–18)
O12	35	2975	3339 (12)	3043 (2.3)

[1]Shown are the shortest study durations required to accurately predict state variables, including TBS, and kinetic parameters (see Figure 3A) in theoretical children (C1 – C4), younger adults (Y5 – Y8), and older adults (O9 – O12)] as well as model-predicted TBS using the reference value for DI, 50% of the reference value for DI, and 150% of the reference value for DI. Also shown are values for percent changes (% Δ) in predicted TBS calculated using 50 or 150% of the reference value for DI compared to the reference value, calculated as [(TBS using DI – TBS using 50 or 150% DI) / TBS using DI] × 100. These results are shown graphically in Figure 4; subjects are described in Supplemental Table 1. DI, dietary intake; TBS, total body stores.

Supplemental Table 4 Reference values for the RID coefficient *FaS* at 4, 7, and 14 d compared with model calculations done without and with dietary vitamin A intake data included in the model[1]

	FaS 4d			*FaS* 7 d			*FaS* 14 d		
Subject	Reference	−DI (% Δ)	+DI (% Δ)	Reference	−DI (% Δ)	+DI (% Δ)	Reference	−DI (% Δ)	+DI (% Δ)
C1	2.71	1.94 (–28)	2.67 (–1.5)	1.43	1.01 (–29)	1.40 (–2.1)	0.532	0.391 (–26)	0.523 (–1.7)
C2	4.14	2.66 (–36)	4.07 (–1.7)	1.89	1.21 (–36)	1.85 (–2.1)	0.722	0.482 (–33)	0.717 (–0.7)
C3	1.78	1.85 (3.9)	1.77 (–0.6)	0.926	0.965 (4.2)	0.925 (–0.1)	0.714	0.739 (3.5)	0.715 (0.1)
C4	2.39	1.31 (–45)	2.34 (–2.1)	1.45	0.773 (–47)	1.42 (–2.1)	0.788	0.454 (–42)	0.777 (–1.4)
**GM model**	**2.67**	**1.65 (–38)**	**2.95 (10)**	**1.40**	**0.849 (–39)**	**1.53 (9.3)**	**0.661**	**0.438 (–34)**	**0.729 (10)**
Y5	1.41	1.11 (–21)	1.41 (0.0)	1.23	0.964 (–22)	1.24 (0.8)	0.977	0.761 (–22)	0.981 (0.4)
Y6	1.45	1.45 (0.0)	1.48 (2.1)	0.838	0.833 (0.6)	0.843 (0.6)	0.649	0.646 (–0.5)	0.652 (0.5)
Y7	2.07	1.59 (–23)	2.09 (1.0)	1.40	1.10 (–21)	1.45 (3.6)	1.00	0.764 (–24)	1.03 (3.0)
Y8	2.13	1.89 (–11)	2.14 (0.5)	1.48	1.31 (–11)	1.48 (0.0)	1.03	0.907 (–12)	1.04 (1.0)
**GM model**	**1.65**	**1.32 (–20)**	**1.68 (1.8)**	**1.23**	**0.953 (–22)**	**1.24 (0.8)**	**0.912**	**0.691 (–24)**	**0.920 (0.9)**
O9	2.54	2.23 (–12)	2.36 (–7.1)	1.57	1.37 (–13)	1.45 (–7.6)	0.829	0.763 (–8.0)	0.795 (–4.1)
O10	2.06	2.18 (5.8)	2.08 (1.0)	1.23	1.30 (5.7)	1.24 (0.8)	0.936	0.994 (6.2)	0.943 (0.7)
O11	3.68	3.3 (–10)	3.69 (0.3)	2.16	1.92 (–11)	2.16 (0.0)	0.972	0.860 (–12)	0.968 (–0.4)
O12	4.48	2.96 (–34)	4.50 (0.4)	2.20	1.45 (–34)	2.21 (0.4)	1.44	0.943 (–34)	1.44 (0.0)
**GM model**	**2.84**	**2.26 (–20)**	**2.90 (2.1)**	**1.63**	**1.29 (–21)**	**1.67 (2.4)**	**1.01**	**0.778 (–23)**	**1.03 (2.0)**

[1]Shown are individual reference values and geometric mean (population) model-predicted values for *FaS* at 4, 7, and 14 d calculated after modeling plasma retinol kinetic data from 3 h to 365 d ("Reference") for 12 theoretical subjects [children (C1 – C4), younger adults (Y5 – Y8), and older adults (O9 – O12)] as well as values calculated using data modeled to 28 d for the children and to 56 d for the adults, without (−DI) and with inclusion of dietary intake data during modeling (+DI). Population modeling was done using geometric mean plasma retinol kinetic data calculated for each of the 3 groups of subjects; compare values to the geometric mean of the individual model-predicted *FaS* at 4, 7, and 14 d for the group of children (2.63, 1.38, and 0.682), younger adults (1.73, 1.21, and 0.899), and older adults (3.05, 1.74, and 1.02). Also shown are values for percent change (% Δ) in *FaS* predicted without or with DI compared to the reference value, calculated as [(reference value – value predicted without or with DI) / reference value] × 100. For the RID coefficient *FaS*, *Fa* is the fraction of the oral vitamin A dose found in the body's extravascular vitamin A pool at 4, 7, or 14 d (fraction of the dose in compartments 6 plus 7; Figure 1) and *S* is retinol specific activity in plasma divided by that in stores (specific activity in compartment 5 / specific activity in compartments 6 plus 7) at that time. Subjects are described in Supplemental Table 1. RID, retinol isotope dilution; DI, dietary intake; GM, geometric mean.

Supplemental Figure 1

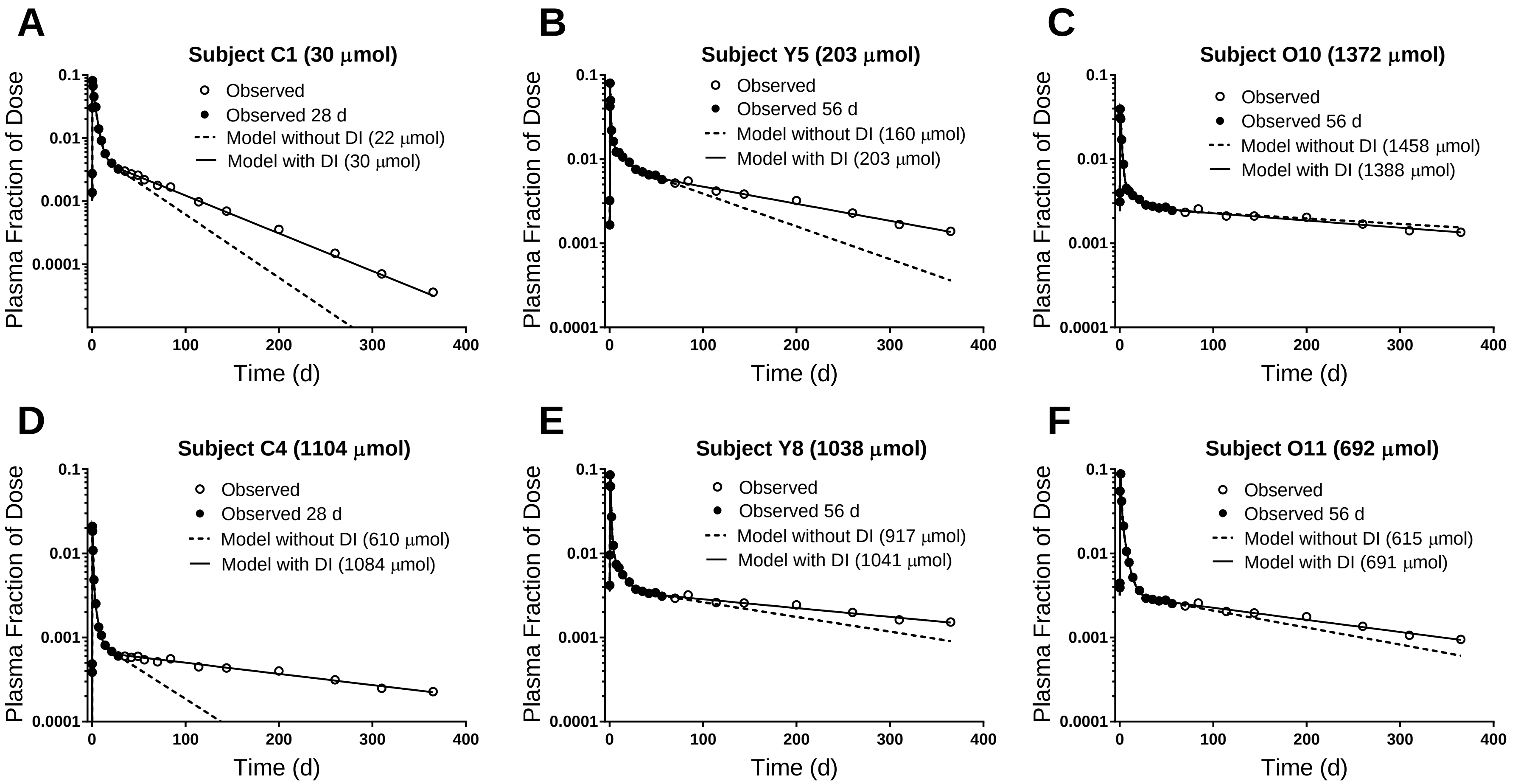

Supplemental Figure 1. Theoretical observed plasma retinol tracer response data versus time and model-calculated curves without and with the addition of dietary vitamin A intake as an input during compartmental modeling for 2 theoretical children [C1 (A) and C4 (D); Supplemental Table 1], 2 younger adults [Y5 (B) and Y8 (E)], and 2 older adults [O10 (C) and O11 (F)]. Shown are data to 365 d as well as the model fits to durations of 28 d for children and 56 d for adults without and with intake included. Reference values for TBS (μmol) are indicated as are TBS predictions without and with intake included. DI, dietary intake; TBS, total body stores.

Supplemental Figure 2

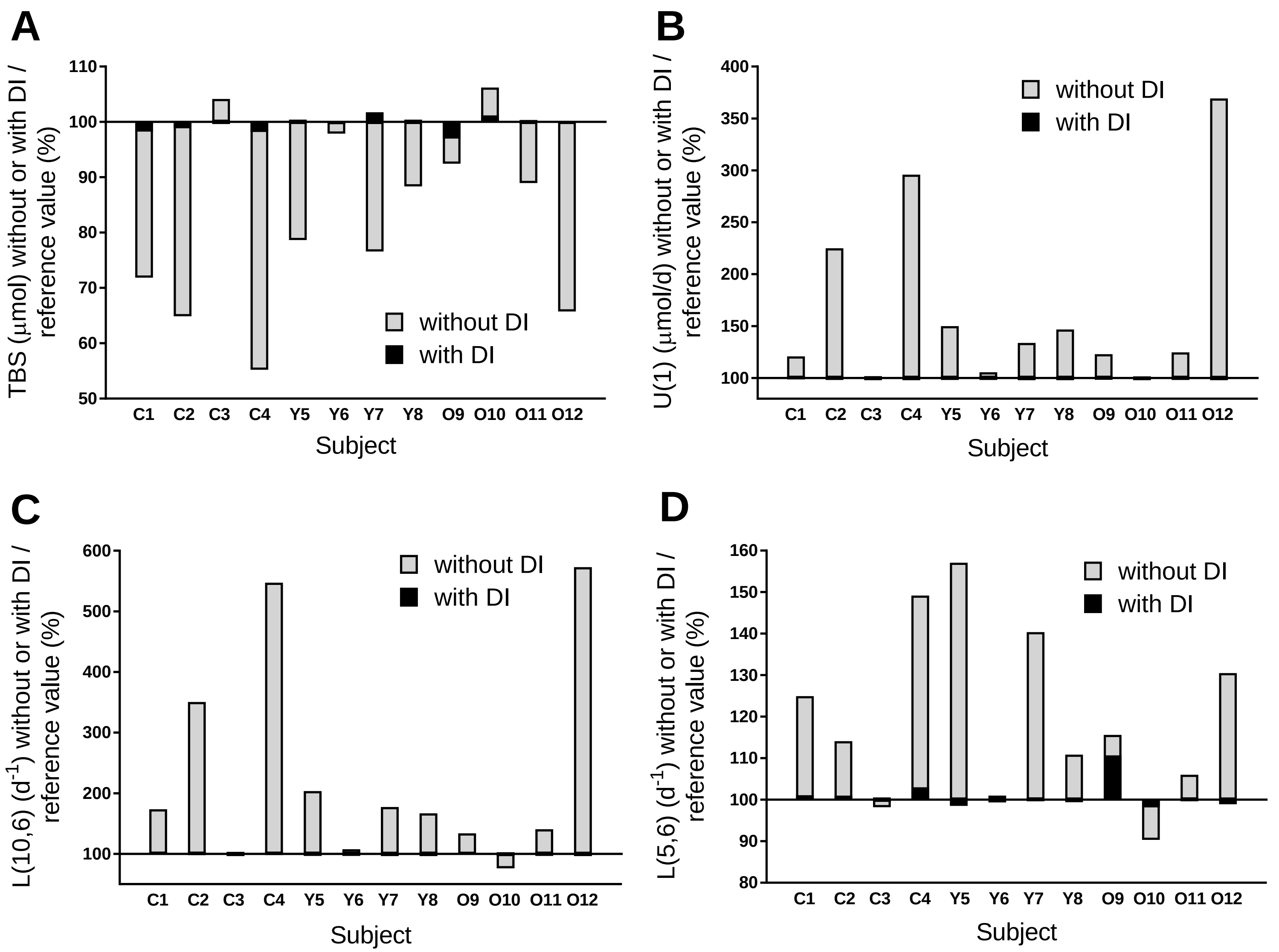

Supplemental Figure 2. Predicted values for the 2 state variables TBS (A) and model-predicted vitamin A intake [U(1), Figure 1; B] and for 2 retinol kinetic parameters, L(10,6) (C) and L(5,6) (D), as percent of reference values for 12 theoretical human subjects. Predicted values were calculated without and with dietary vitamin A intake included during modeling of data to 28 d for the 4 theoretical children (C1 – C4) and to 56 d for the younger (Y5 – Y8) and older adults (O9 – O12); reference values were obtained from modeling data to 365 d (Supplemental Table 1). DI, dietary intake; TBS, total body stores.

Supplemental WinSAAM Deck

```
A SAAM31          Y7 2EV FFE 114D        [4-JAN-19]
CC YOUNGER ADULT; 22 YR; [ROH] = 1.4 UMOL/L; INTAKE = 1100 UG RAE/D; BW = 60 KG
CC 7-COMPARTMENT MODEL WITH LOSS FROM ONLY COMPARTMENT 6
CC 2 EXTRAVASCULAR POOLS: COMPARTMENT 6 (LARGER) AND COMPARTMENT 7 (SMALLER)
CC FIXED FRONT END: FIXED L(2,1) AND L(5,2) AT REFERENCE VALUES
CC PARAMETERS
H PAR
CC          VALUE          LOWER LIMIT    UPPER LIMIT
CC IC(I)=INITIAL CONDITION (FRACTION OF DOSE) IN COMPARTMENT I AT TIME 0
   IC(1)=1
CC L(I,J)=FRACTION OF J TRANSFERRED TO I PER DAY (DAY^-1)
   L(2,1)    6.052309E+01
CC L(0,1)=FRACTIONAL LOSS OF UNABSORBED TRACER
CC ASSUMING 75% ABSORPTION EFFICIENCY; L(0,1)=1/3 OF L(2,1)
   L(0,1)=0.333*L(2,1)
   L(3,2)=L(2,1)
   L(5,2)    8.232734E-01
CC DN(I)=NUMBER OF ELEMENTS IN DELAY COMPONENT I
CC DT(I)=DELAY TIME IN COMPONENT I (DAY)
   DN(3)    8
   DT(3)     1.280575E-01  0.000000E+00   1.000000E+02
CC OUTPUT FROM DELAY COMPONENT EQUALS 1
   L(4,3)=1
   L(5,4)    9.624985E-01  0.000000E+00   1.000000E+02
   L(6,5)    2.934716E+00  0.000000E+000  1.000000E+02
   L(5,6)    1.612310E-02  0.000000E+00   1.000000E+02
CC L(10,6)=FRACTIONAL CATABOLIC RATE OF COMPARTMENT 6;
CC COMPARTMENT 10 IS LOSS FROM THE SYSTEM
   L(10,6)   6.692401E-03  0.000000E+00   1.000000E+02
   L(7,5)    4.131773E+00  0.000000E+00   1.000000E+02
   L(5,7)    1.934754E-01  0.000000E+0000 1.000000E+02
CC STEADY STATE SOLUTION
CC U(I)=VITAMIN A INTAKE RATE (UMOL/D)
CC M(I)=MASS OF VITAMIN A IN COMPARTMENT I (UMOL)
H STE
C REFERENCE VALUE FOR DIETARY INTAKE = 3.99 UMOL RAE/D
   U(1)      4.188332E+00 0             100
CC M(5)=PLASMA RETINOL POOL (UMOL)
   M(5)     3.65
CC MODEL-PREDICTED MASSES IN COMPARTMENTS 6 & 7
CC M(6)=469 UMOL
CC M(7)=78 UMOL
CC DATA
H DAT
CC PLASMA RETINOL FRACTION OF TRACER DOSE
CC THEORETICAL OBSERVED DATA WITH ERROR ADDED
CC                                      FRACTIONAL STANDARD DEVIATION
105                                     FSD=0.05
CC          TIME (D)      FRACION OF DOSE

            0             0
            0.125         0.00504731
            0.208         0.0320467
            0.333         0.0696177
```

```
           0.5          0.0758015
           1            0.0564934
           2            0.028841
           4            0.0146723
           7            0.00852208
           10           0.00790725
           14           0.00657486
           21           0.00527302
           28           0.0041223
           35           0.00370767
           42           0.00333708
           49           0.00326275
           56           0.00286729
           70           0.00256802
           84           0.002692
           114          0.00200409
CC TIMES EXCLUDED
CC MINIMUM TIME FOR ACCURATE PREDICTION OF VITAMIN A STORES [M(6)+M(7)] AND
CC KINETIC PARAMETERS WITHOUT DIETARY INTAKE INCLUDED DURING MODELING WAS 114 DAY
Y
           144          0.00181444
           200          0.00146703
           260          0.00100103
           310          0.000708661
           365          0.000567534
Y-
CC PLASMA SIMULATIONS
105
           0
2          0.05                          20
2          0.1                           40
2          1                             60
2          10                            30
```

A SAAM31 Y7 2EV FFE 28D DI [4-JAN-19]

```
CC YOUNGER ADULT; 22 YR; [ROH] = 1.4 UMOL/L; INTAKE = 1100 UG RAE/D; BW = 60 KG
CC 7-COMPARTMENT MODEL WITH LOSS FROM ONLY COMPARTMENT 6
CC 2 EXTRAVASCULAR POOLS: COMPARTMENT 6 (LARGER) AND COMPARTMENT 7 (SMALLER)
CC FIXED FRONT END: FIXED L(2,1) AND L(5,2) AT REFERENCE VALUES
CC REFERENCE VALUE FOR DIETARY VITAMIN A INTAKE INCLUDED
CC PARAMETERS
H PAR
CC         VALUE        LOWER LIMIT    UPPER LIMIT
CC IC(I)=INITIAL CONDITION (FRACTION OF DOSE) IN COMPARTMENT I AT TIME 0
   IC(1)=1
CC L(I,J)=FRACTION OF J TRANSFERRED TO I PER DAY (DAY^-1)
   L(2,1)    6.052309E+01
CC L(0,1)=FRACTIONAL LOSS OF UNABSORBED TRACER
CC ASSUMING 75% ABSORPTION EFFICIENCY; L(0,1)=1/3 OF L(2,1)
   L(0,1)=0.333*L(2,1)
   L(3,2)=L(2,1)
   L(5,2)    8.232734E-01
CC DN(I)=NUMBER OF ELEMENTS IN DELAY COMPONENT I
CC DT(I)=DELAY TIME IN COMPONENT I (DAY)
   DN(3)     8
   DT(3)     1.277356E-01  0.000000E+00   1.000000E+02
```

```
CC OUTPUT FROM DELAY COMPONENT EQUALS 1
   L(4,3)=1
   L(5,4)    9.566745E-01  0.000000E+00   1.000000E+02
   L(6,5)    2.954797E+00  0.000000E+000  1.000000E+02
   L(5,6)    1.620557E-02  0.000000E+00   1.000000E+02
CC L(10,6)=FRACTIONAL CATABOLIC RATE OF COMPARTMENT 6;
CC COMPARTMENT 10 IS LOSS FROM THE SYSTEM
   L(10,6)   6.232547E-03  0.000000E+00   1.000000E+02
   L(7,5)    4.104903E+00  0.000000E+00   1.000000E+02
   L(5,7)    1.924474E-01  0.000000E+0000 1.000000E+02
CC STEADY STATE SOLUTION
CC U(I)=VITAMIN A INTAKE RATE (UMOL/D)
CC M(I)=MASS OF VITAMIN A IN COMPARTMENT I (UMOL)
H STE
C REFERENCE VALUE FOR DIETARY INTAKE = 3.99 UMOL RAE/D
   U(1)      3.993281E+00 0             100
CC M(5)=PLASMA RETINOL POOL (UMOL)
   M(5)     3.65
CC MODEL-PREDICTED MASSES IN COMPARTMENTS 6 & 7
CC M(6)=481 UMOL
CC M(7)=78 UMOL
CC DATA
H DAT
CC WEIGHTED U(1) AT REFERENCE VALUE FOR DIETARY VITAMIN A INTAKE
CC          TIME (D)      U(1)          FRACTIONAL STANDARD DEVIATION
100                                     FSD=0.05
   U(1)     0             3.99
CC PLASMA RETINOL FRACTION OF TRACER DOSE
CC THEORETICAL OBSERVED DATA WITH ERROR ADDED
CC                                      FRACTIONAL STANDARD DEVIATION
105                                     FSD=0.05
CC          TIME (D)      FRACION OF DOSE
            0             0
            0.125         0.00504731
            0.208         0.0320467
            0.333         0.0696177
            0.5           0.0758015
            1             0.0564934
            2             0.028841
            4             0.0146723
            7             0.00852208
            10            0.00790725
            14            0.00657486
            21            0.00527302
            28            0.0041223
CC TIMES EXCLUDED
CC MINIMUM TIME FOR ACCURATE PREDICTION OF VITAMIN A STORES [M(6)+M(7)] AND
CC KINETIC PARAMETERS WITH DIETARY INTAKE INCLUDED DURING MODELING WAS 28 DAY
Y
            35            0.00370767
            42            0.00333708
            49            0.00326275
            56            0.00286729
            70            0.00256802
            84            0.002692
            114           0.00200409
```

	144	0.00181444
	200	0.00146703
	260	0.00100103
	310	0.000708661
	365	0.000567534

Y-
CC PLASMA SIMULATIONS

105

	0	
2	0.05	20
2	0.1	40
2	1	60
2	10	30

Supplemental References

1. Linderkamp O, Versmold HT, Riegel KP, Betke K. Estimation and prediction of blood volume in infants and children. Eur J Pediatr 1977;125:227-34.
2. Hahn RG. Blood volumes and fluid kinetics. In: Hahn RG, editor. Clinical fluid therapy in the perioperative setting. Cambridge (UK): Cambridge University Press; 2011. p. 127 – 36.
3. Green MH, Green JB. Vitamin A intake and status influence retinol balance, utilization and dynamics in rats. J Nutr 1994;124:2477-85.
4. Oxley A, Berry P, Taylor GA, Cowell J, Hall MJ, Hesketh J, Lietz G, Boddy AV. An LC/MS/MS method for stable isotope dilution studies of β-carotene bioavailability, bioconversion, and vitamin A status in humans. J Lipid Res 2014;55:319-28.
